# An Exercise-Based Precision Medicine Tool and Smartphone App for Managing Achilles Tendinopathy (the 'PhysViz' System): User-Centered Development Study

**DOI:** 10.2196/57873

**Published:** 2024-11-13

**Authors:** Kohle Merry, Megan M MacPherson, Jackie L Whittaker, Christopher Napier, Liisa Holsti, Alex Scott

**Affiliations:** 1 Department of Physical Therapy University of British Columbia Vancouver, BC Canada; 2 Virtual Health Department Fraser Health Authority Surrey, BC Canada; 3 Arthritis Research Canada Vancouver, BC Canada; 4 Department of Biomedical Physiology and Kinesiology Simon Fraser University Burnaby, BC Canada; 5 Department of Occupational Science and Occupational Therapy University of British Columbia Vancouver, BC Canada

**Keywords:** exercise therapy, physical therapy modalities, rehabilitation, tendons, tendinopathy, mobile health, mHealth, mobile phone

## Abstract

**Background:**

People with Achilles tendinopathy (AT) experience persistent pain that can limit engagement with daily occupations and negatively impact mental health. Current therapeutic exercise approaches vary in success, with many people experiencing reinjury, leading to a cycle of chronic tendinopathy often lasting years. High-magnitude precision loading may help people exit this feedback cycle, but applying these principles clinically is challenging.

**Objective:**

This user-centered design case study aims to provide an overview on how the PhysViz (a prototype for a novel remote rehabilitation intervention for AT management) was developed and evaluated following the development phase of the Framework for Accelerated and Systematic Technology-Based Intervention Development and Evaluation Research (FASTER).

**Methods:**

The development process engaged a multidisciplinary team comprising people with AT experiences, clinicians, and engineers. It followed the 5 stages within the FASTER development phase: empathize, define, ideate, prototype, and test. The PhysViz development and evaluation were informed by needs assessments, surveys, literature reviews, validation studies, case studies, roundtable discussions, and usability testing (some of which have been published previously). The FASTER systematically guided the integration of evidence-based features and behavior change theory.

**Results:**

By using the FASTER and ensuring that the PhysViz system was underpinned by diverse stakeholder needs, this work resulted in the development of a working prototype for both the PhysViz physical exercise tool and the accompanying PhysViz software package (mobile app and web application). A variety of study designs informed user-desired features that were integrated into the PhysViz prototype, including real-time biofeedback in the form of precision load monitoring, customizable exercise programs, and pain tracking. In addition, clinicians can visualize client data longitudinally and make changes to client exercise prescriptions remotely based on objective data. The identified areas for improvement, such as upgrading the user interface and user experience and expanding clinical applications, provide valuable insights for future PhysViz iterations. Further research is warranted to assess the long-term efficacy and feasibility of the PhysViz in diverse clinical settings and its potential to improve AT symptoms.

**Conclusions:**

Being one of the first technology development initiatives guided by the FASTER, this study exemplifies a systematic and multidisciplinary approach to creating a remote rehabilitation intervention. By incorporating stakeholder feedback and evidence-based features, the PhysViz addresses key challenges in AT rehabilitation, offering a novel solution for precision loading and therapeutic exercise engagement. Positive feedback from users and clinicians underscores the potential impact of the PhysViz in improving AT management outcomes. The PhysViz serves as a model for technology-based intervention development, with potential implications for other tendinopathies and remote rehabilitation strategies.

## Introduction

### Background

Achilles tendinopathy (AT)—a chronic condition characterized by ongoing pain in the Achilles tendon and loss of function related to mechanical loading [1]—may limit participation in daily activities while negatively impacting mental health and reducing quality of life [2]. Although therapeutic exercise is the cornerstone of nonsurgical AT management [3-5], success varies [6-9], and rates of reinjury are high (eg, up to 27% in elite athletes) [10]. Tendinopathy may be recalcitrant to treatment [11,12] due, in part, to inappropriate treatment selection, inadequate dosing of therapeutic exercises, or psychosocial or contextual factors that are barriers to therapeutic exercise adherence [13,14]. Taken together, these facts highlight the need for research to optimize exercise-based AT treatment protocols and methods to personalize and deliver these protocols to promote adherence to therapeutic exercise and improve clinical outcomes.

Therapeutic exercise using high-magnitude loading (ie, >70% of maximal voluntary contraction [MVC]) can elicit positive adaptation in healthy tendons [15,16]. The extent to which these findings transfer to tendinopathic tendons remains largely unknown [17]. An individualized dose of 70% MVC exercise is higher than what is typically used in AT rehabilitation [6,18-20], and clinicians such as physical therapists (PTs) may struggle to achieve both this load magnitude and precision in the clinic. Although a 70% MVC prescription may be achieved clinically by basing an exercise load on a patient’s 1-repetition maximum, remote monitoring systems may improve access to adjustable high-magnitude precision loading and promote exercise adherence.

Remote monitoring systems can improve access to care, reduce the need for in-person follow-up and associated costs, and increase patient engagement with self-monitoring and built-in opportunities for clinician feedback [21,22]. As pain behavior is 1 method clinicians use to moderate AT exercise programs [6,18-20] (eg, instructions to not exceed a certain rating on a 10-point numerical pain rating scale during therapeutic exercises), home-based precision exercise dosing incorporating biofeedback and remote clinician monitoring could provide patients reassurance that their pain is acceptable and that they are operating in the correct loading range.

Existing dosing feedback systems are not yet practical or scalable due to prohibitive costs, space requirements (ie, isokinetic dynamometers), functional limitations (eg, positioning using handheld dynamometry as well as appropriate biofeedback and user interface [UI] and user experience [UX]) [23,24], and a lack of tailoring for home use by nonprofessionals. Given these limitations, and within the scope of AT rehabilitation, the development of a new system to facilitate precision dose–based therapeutic exercise is warranted. Knowing that the magnitude and precision of loading are important factors for inducing positive tendon adaptation [15,16], a home-based exercise-dosing system incorporating biofeedback and remote monitoring could potentially improve outcomes for those with AT. This paper provides a high-level overview of the development of 1 such system using the Framework for Accelerated and Systematic Technology-Based Intervention Development and Evaluation Research (FASTER) [25].

### Prior Work

The concept for a home-based training system to enable high-magnitude loading of the triceps surae (ie, the gastrocnemius, soleus, and plantaris muscles, which act through the Achilles tendon to plantarflex the foot) comes from researchers at the Institute of Sport Sciences at the Humboldt University of Berlin [26]. In 2020, the Tendon Injury Prevention and Rehabilitation Group at the University of British Columbia consulted with the Humboldt group and obtained permission to adapt the Humboldt researchers’ idea for the development of an AT remote rehabilitation system, called the 'PhysViz'.

The PhysViz comprises a physical exercise tool incorporating a Bluetooth-enabled load sensor and the PhysViz software package consisting of a patient-facing mobile phone app and clinician-facing web application. The purpose of the PhysViz is to facilitate high-magnitude loading of the Achilles tendon, collect loading data, provide real-time biofeedback during loading to promote exercise engagement, centralize all patient data for review, and enable the remote modification of a patient’s exercise prescription by an overseeing clinician. The goal of the system is to enhance AT rehabilitation through high-load precision exercise–dosing approaches and empower active participation of patients in their rehabilitation through the dissemination of actionable information to both patients and clinicians.

### The Goal of This Study

Designing a new system to facilitate precision dose–based therapeutic exercise in an evidence-based and user-informed manner is challenging. Traditional design often prioritizes time to market, potentially at the expense of rigor [27]. Rigorous, evidence-based, user-centric development takes time; however, such a time delay may lead to technologies becoming obsolete, implementation conditions changing, or new interventions emerging [25]. Unfortunately, a significant portion of medical research funding (up to 80%) ends up as “research waste,” failing to make a notable impact on public health [28]. In addition, 50% of clinical innovations never reach widespread clinical adoption [29], and the translation of research-based solutions into clinical practice can take upwards of 17 years [30]. As such, technology-based clinical interventions must strategically develop solutions and generate supporting evidence in a timely manner [25]. This work aims to describe the approach taken to develop the PhysViz, which features user-centric development and theoretical underpinning. This manuscript not only presents the systematic approach taken in developing the PhysViz system but also highlights both previously published findings (primarily in the empathize stage within the FASTER development phase) and new findings (primarily in the prototype and test stages).

## Methods

### Design Approach

To promote the timely and strategic generation of supporting evidence, the PhysViz development is guided by the FASTER [25]. The FASTER includes 3 phases: development, progressive usability and feasibility evaluation, and scaled evaluation and implementation. This work is situated within the development phase, which aims to engage with end users and use transdisciplinary thinking (eg, engineering design and clinical experience) to generate actionable evidence appropriate for the efficient design and implementation of technology-based interventions. Specifically, within this phase, the FASTER underscores the importance of empathizing with users to understand their needs, preferences, and experiences while also using established design theories to generate ideas, develop prototypes, test them, and iterate. The FASTER development phase can be further broken down into 5 stages derived from Stanford Design Thinking: empathize, define, ideate, prototype, and test [31].

**Figure 1:**
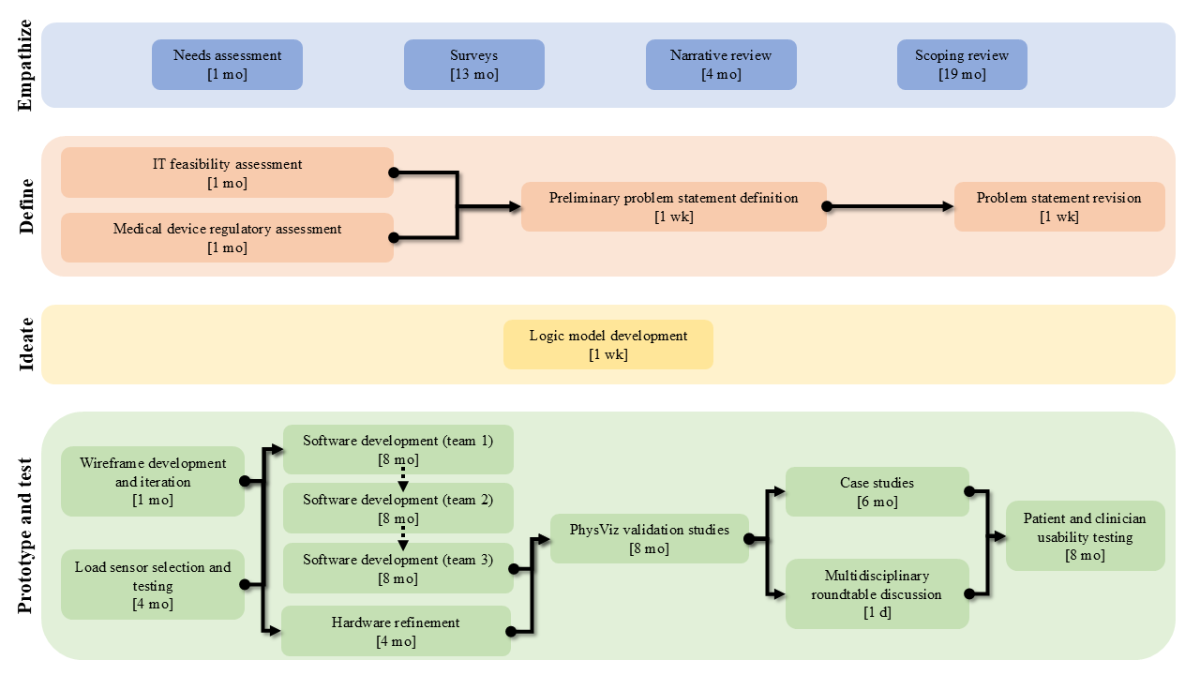
PhysViz development timeline according to the development phase of the Framework for Accelerated and Systematic Technology-Based Intervention Development and Evaluation Research. The arrows denote the progression of tasks. When arrows are not present, this denotes tasks completed concurrently. Development broadly progressed from the empathize stage to the prototype and test stages.

This work describes 1 of the first case studies applying the FASTER for the development of a remote rehabilitation system. An overview of the design approach and timeline is presented in Figure 1. By integrating previously published studies with new research, this manuscript offers a comprehensive perspective on the PhysViz development process guided by the FASTER. It is important to note that this manuscript offers a broad overview of how various research studies contributed to the PhysViz design through the FASTER development phase. It does not provide a detailed account of the methods and results of each individual study involved in the PhysViz development. To ensure transparency and facilitate replication of the development process, studies that have not been previously published in peer-reviewed journals have been deposited on the Open Science Framework website [32-34].

### Stages of the FASTER Development Phase

#### Empathize

Within the FASTER empathize stage, researchers should determine end-user needs and preferences while also examining the broader context to define the research problem in detail [25]. As such, this stage consists of a needs assessment and surveys to garner information on end-user needs, while also including both narrative and scoping reviews to gain a broader understanding of exercise-based AT rehabilitation.

##### Needs Assessment

To assess end-user needs for the patient-facing PhysViz mobile app, our research team completed a needs assessment featuring clinicians, engineers, patient partners, and researchers. Due to the transdisciplinary nature of developing a software tool for home-based use by patients, it was vital to get multiple perspectives to establish the initial direction of the app development. Specifically, the research team was asked the following in reference to the PhysViz app: (1) what features they must see; (2) what features they would like to see; (3) what information they would like tracked; (4) what they would like the UI to look like; (5) whether they expected to see an overview or summary dashboard, and if so, what information they would like displayed there; (6) what type of security protection they expected to see; and (7) whether they had any other comments or relevant information pertaining to the development. All suggestions were collated and presented back to the research team for communal ranking before starting the development process.

##### Surveys

As described in Merry et al [35], to further identify end-user needs and preferences within the specific implementation context, we conducted 2 surveys to identify perceived barriers and facilitators to participating in and prescribing exercise-based therapies for AT among people with AT and PTs, respectively. While the PT survey assessed both clinical practice patterns and barriers and facilitators to developing, prescribing, and monitoring exercise-based treatments for AT, the survey of people with AT expanded upon the barriers and facilitators pertaining to engagement and adherence to exercise-based rehabilitation for AT through a series of questions using the capability, opportunity, motivation, and behavior (COM-B) model [36,37]. According to the COM-B model, for a behavior to occur, an individual must have sufficient *capability* (physical and psychological), *opportunity* (social and physical), and *motivation* (automatic and reflective). In addition, we asked participants what potential app features are desirable in the context of AT rehabilitation.

##### Narrative and Scoping Reviews

As described in Merry et al [17], we completed a narrative review to provide an overview of the broader AT context by (1) synthesizing the principles of tendon remodeling under resistance exercise–induced loading among both healthy and pathological tendons and (2) commenting on the biomechanical principles of Achilles tendon loading that may impact a therapeutic exercise prescription for AT. This work not only served to identify research gaps and potential future use cases for the PhysViz, but it was also undertaken to inform aspects of the PhysViz development based on prior evidence.

As described in Merry et al [32,38] and in parallel to the narrative review, we conducted a scoping review to synthesize how current resistance exercise–based AT interventions are being designed and implemented clinically. Given that therapeutic exercise is considered a primary clinical management strategy for AT [3,4], this review sought to understand what aspects of therapeutic exercise may be common across the clinical research evidence (in contrast to the largely mechanistic evidence encompassed in the narrative review) and how these aspects could potentially be built into the PhysViz design [32,38].

#### Define

In the define stage, information from the empathize stage is used to specify the problem in detail, including its location, the potential users and stakeholders involved, and any other pertinent details. Using both the contextual and research evidence identified in the empathize stage, we defined a preliminary problem statement. The problem statement was then refined after a medical device regulatory assessment and an IT feasibility assessment.

#### Ideate

The ideate stage then focuses on the generation of ideas and the exploration of possible solutions to the problem. Specifically, we developed a logic model for the PhysViz based on the therapeutic exercise treatment principles (both mechanistic and clinical) identified in the empathize stage coupled with the barriers and facilitators to participating in and prescribing therapeutic exercise for AT identified using the COM-B model. The purpose of developing the logic model was to link potential PhysViz features to desirable behavioral and treatment outcomes a priori; in this way, features could be strategically developed based on evidence and proposed treatment pathways rather than on personal opinions or “it sounded like a good idea at the time” logic.

#### Prototype and Test

##### Overview

The prototype stage describes the creation of low-fidelity prototypes using select ideas from the ideate stage to help the team members visualize and communicate their ideas. Select prototypes are then advanced to the test stage where feedback from end users and stakeholders is garnered to evaluate the feasibility of the design solution. The prototype and test stages are critical for ensuring that the team’s design solution is feasible and meets the needs of the end users. The iterative nature of these stages allows for continual improvements and adjustments, ultimately leading to the timely development of a more appropriate and user-friendly final product.

Using the logic models as a feature guide, we developed a series of wireframe designs for the PhysViz patient-facing mobile app and clinician-facing web application. The wireframes were then presented to the research team for review and iteration before beginning the software development. We completed the development of the PhysViz software consisting of both a mobile app and a web application in collaboration with 3 teams of senior computer engineering students from the University of British Columbia. Each team, consisting of 4 to 5 students, completed 8 months of development before passing the development to the next team. Building upon the initial design developed by the Humboldt group [26] and given the incorporation of a Bluetooth-enabled load sensor in the PhysViz design, the first PhysViz physical exercise tool was tested by the research team to assess viability and potential modifications based on learnings from the empathize stage.

##### Load Sensor Selection and Testing

As described in Merry et al [39], a fundamental aspect of the PhysViz design was the inclusion of a Bluetooth-enabled load sensor enabling mobile phone connectivity with the physical exercise tool. To start, we searched for an appropriate load sensor to meet the following criteria: (1) the product must be Bluetooth ready, (2) both tensile and compressive sensors were considered, (3) there were no restrictions on price, and (4) high-load tensile load sensors (eg, crane scales) were not considered. We reviewed 11 load sensors and selected 2 for more rigorous testing based on relevant technical specifications. Advanced testing consisted of comparing each load cell’s output against a gold standard mechanical testing machine to assess validity and reliability [39]. After the completion of this study, a single load sensor was selected for inclusion in the physical exercise tool.

##### PhysViz Validation

In conjunction with work undertaken by Pratt et al [40], with an initial prototype of the PhysViz system complete and before using it for AT rehabilitation purposes, it was important to determine whether the system could consistently and accurately measure in vivo muscle strength compared to computerized dynamometry, which is the gold standard for assessing muscle function; for example, if a particular exercise prescription was meant to be based on an individual’s MVC, was the MVC recorded using the PhysViz valid and reliable, thus promoting safe use among individuals who were symptomatic? We completed a validation study with healthy individuals to establish the efficacy of the PhysViz as a potential home-based exercise tool [40].

In conjunction with work undertaken by Schreiber et al [33], given the fundamental roles of precision load management and objective feedback (via the app) in the PhysViz system, it was also important to quantify whether real-time biofeedback has the potential to enhance the execution of AT rehabilitation exercises; for example, if an exercise program prescribed isometric plantar flexion exercises at 70% of an individual’s MVC, would the biofeedback provided by the PhysViz to the individual improve their ability to execute the exercises at the prescribed load? We completed a cross-sectional study with healthy individuals to study the effect of biofeedback within this context [33].

##### Case Studies

Having completed basic validation of the PhysViz system, we conducted three 12-week case studies among people with current AT symptoms under the oversight of a clinician-scientist. Case studies began with an introductory session where we gave each participant basic instructions on how to use the PhysViz. We then tested the participant’s plantarflexion MVC using the PhysViz, which informed the first therapeutic exercise dose intensity (70% of MVC). We asked each participant to exercise 2 to 3 times a week using the “exercise mode” of the PhysViz, which guides users through a therapeutic exercise session using audiovisual biofeedback. The research clinician checked in with participants approximately every 3 weeks and adjusted the therapeutic exercise dose based on participant tolerability. At the end of the 12 weeks, we held a semistructured debrief interview with each participant to discuss intervention acceptability, ergonomics, UI design, and comfort.

##### Multidisciplinary Roundtable Discussion

In parallel to the case studies, we assembled a stakeholder group for an open discussion to (1) refine the design through communal knowledge with a focus on proposals for improvement (customization and flexibility) and utility, (2) consider potential feature additions identified during the survey, and (3) comment on potential use cases beyond AT. The stakeholder group consisted of the research team in addition to external clinician-scientists, practicing clinicians (PTs, occupational therapists, and physicians), people with lived AT experiences, engineers, and insurers or regulators.

##### Patient and Clinician Usability Testing

As described in Merry et al [34], to obtain further user feedback on the PhysViz prototype, we completed a cross-sectional usability study including both people with AT and PTs. We used semistructured 1-on-1 interviews with embedded usability testing featuring a concurrent think-aloud method [41] to inform iteration on the PhysViz system in conjunction with the case studies and roundtable discussion. Combined results from the usability testing and concurrent think-aloud findings were used to create a list of potential design alterations based on common usability issues and end-user suggestions for improvement. The proposed modifications were then evaluated independently by 2 researchers (KM and MMM) using the affordability, practicability, effectiveness, acceptability, side effects and safety, and equity (APEASE) criteria [42]. Interrater agreement was assessed using Cohen’s κ and prevalence-adjusted bias-adjusted κ. Values between 0.61 and 0.80 indicate substantial agreement, and values exceeding 0.80 indicate almost perfect agreement [43,44]. A critical appraisal of each proposed modification using the APEASE criteria facilitated the inclusion of only those modifications deemed most feasible for potential implementation in the second version of the PhysViz system.

### Ethical Considerations

Ethics approval for this study was obtained from the University of British Columbia Clinical Research Ethics Board (approval number: H21-02879). Participants provided informed consent to participate in the study before taking part.

## Results

### Empathize

#### Needs Assessment

Five members of the research team completed the needs assessment, which led to establishing an initial direction for the PhysViz software development. Common “must-see” features included load monitoring and biofeedback, a customizable exercise program, app availability for both iOS and Android devices, and the ability to sync data between the mobile app and the web application. Some examples of “would be nice” features included instructions for participants (eg, video demonstrations or animations), reminders or notifications, a program calendar, and 2-way interaction capabilities between the patient and clinician. A desirable UI was defined by participants as simple and minimalistic, with an emphasis on ease of use for users of different ages and digital literacy levels. User safety (people with AT) was noted as particularly important with a need to avoid placing any patient in a potentially harmful position by trying to complete therapeutic exercises exceeding recommended intensities.

#### Surveys

The survey results, which are published elsewhere [35], led to the following features being prioritized within the initial conceptualization of the PhysViz: exercise demonstrations, education on AT and treatment strategies, guided exercise sessions (ie, automatically counts sets, repetitions, and rest periods), and pop-up reminders for completing the exercise program.

#### Narrative and Scoping Reviews

The narrative review results, which are published elsewhere [17], highlighted the importance of high-load tolerability with the PhysViz design. The long sitting position was identified as advantageous to allow for ankle dorsiflexion and knee extension within the PhysViz design.

The findings from the scoping review, which are published elsewhere [38], emphasized the importance of modifiable exercise programs (both in terms of adjustable resistance and exercise volume) and the incorporation of techniques that allow for improved tracking of treatment fidelity and adherence within the PhysViz design.

### Define

Primary users of the PhysViz system are expected to be people with AT and PTs or other clinicians (eg, occupational therapists and physicians) who oversee and guide AT management. Secondary users will include clinical practice regulators, health care insurers, software engineers, and researchers. The initial problem statement was as follows: “People with chronic AT need a better way to deliver heavy loads to the Achilles tendon because not everyone has access to exercise equipment, and sufficient loading intensity appears to be important for tendon rehabilitation.” After regulatory and feasibility assessments, the problem statement was revised as follows: “People with chronic AT want to get back to their sports and daily occupations quickly but often struggle to adhere to therapeutic exercise programs and load the tendon heavily enough. Emerging exercise technologies have the potential to empower users, providing autonomy and objective feedback, thereby facilitating a quicker return to their activities.”

### Ideate

**Figure 2:**
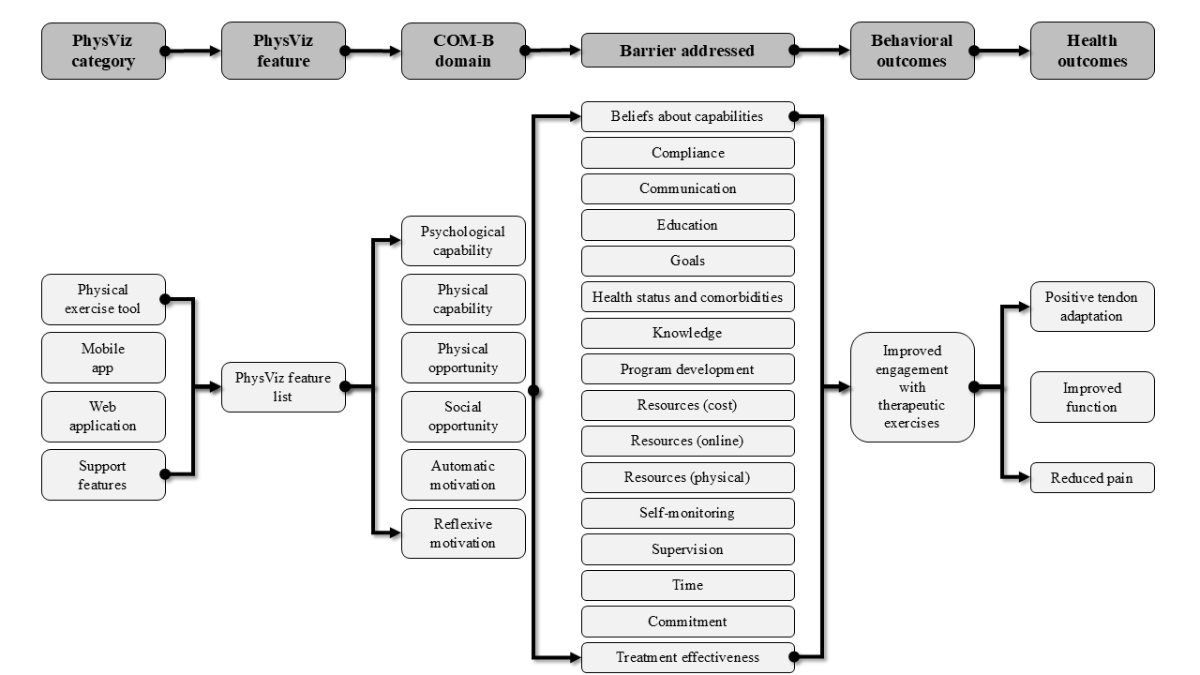
PhysViz development general logic model. COM-B: capability, opportunity, motivation, and behavior.

We used findings from the empathize stage to inform the development of a logic model to address the revised problem statement and guide the initial development of the PhysViz system by linking potential evidence-based features with behavioral and treatment outcomes. Figure 2 presents a general logic model, while detailed logic models are presented in Multimedia Appendix 1.

### Prototype and Test

#### Overview

**Figure 3:**
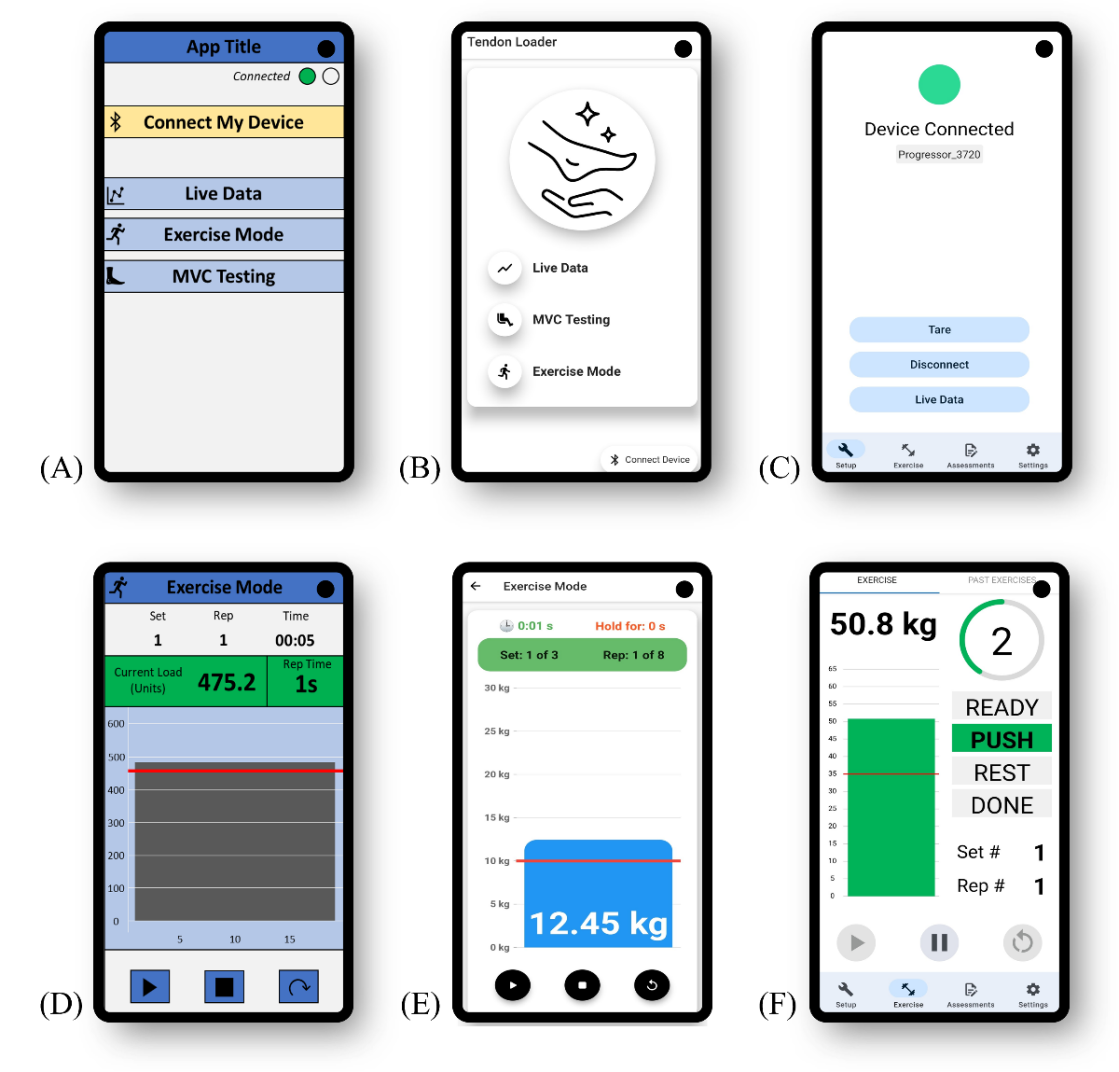
(A-C) PhysViz mobile app home page. (D-F) “Exercise mode” page with real-time biofeedback. (A and D) Initial wireframe. (B and E) First prototype. (C and F) Current prototype.

**Figure 4:**
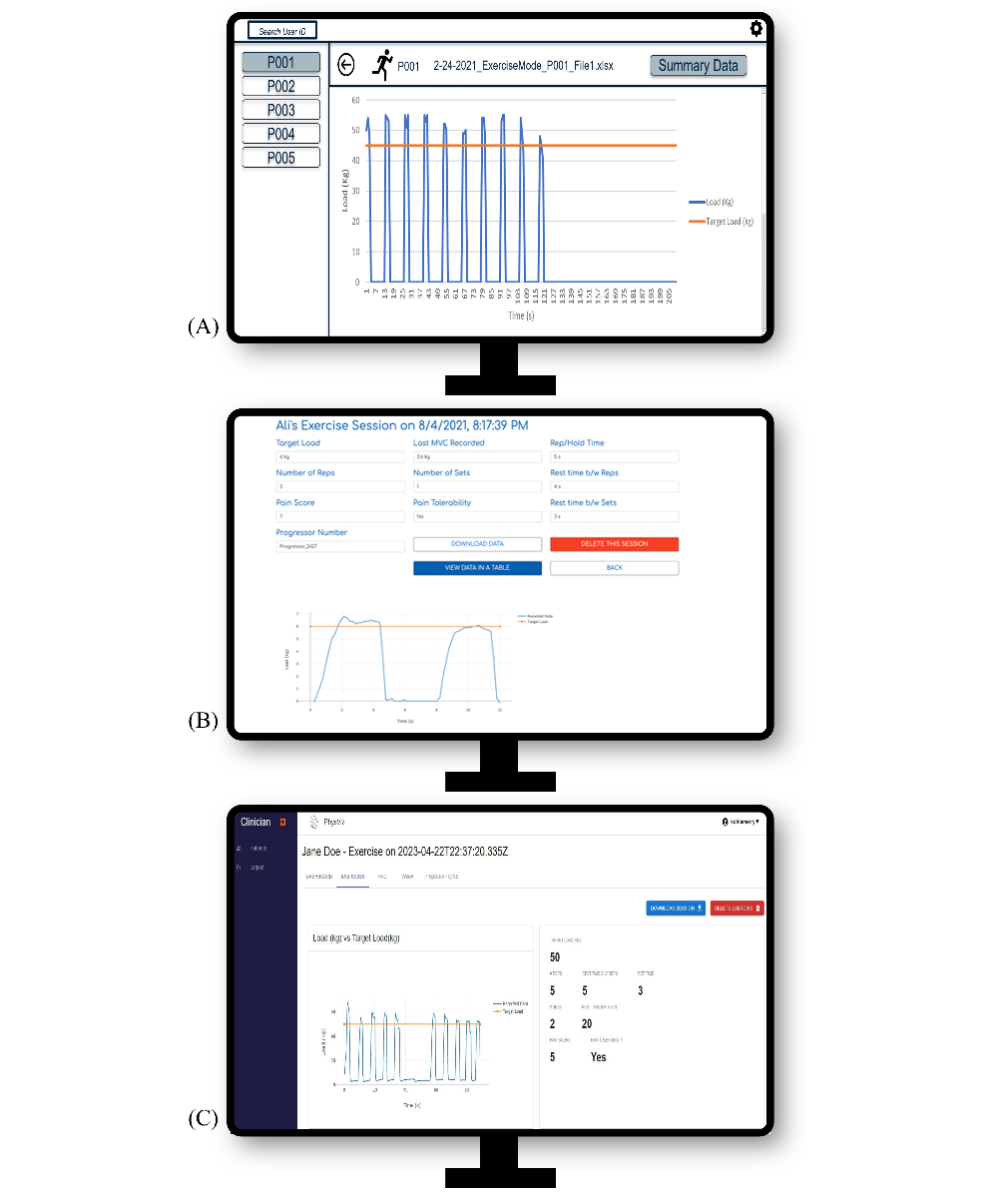
PhysViz web application sample exercise page. (A) Initial wireframe. (B) First prototype. (C) Current prototype.

**Figure 5:**
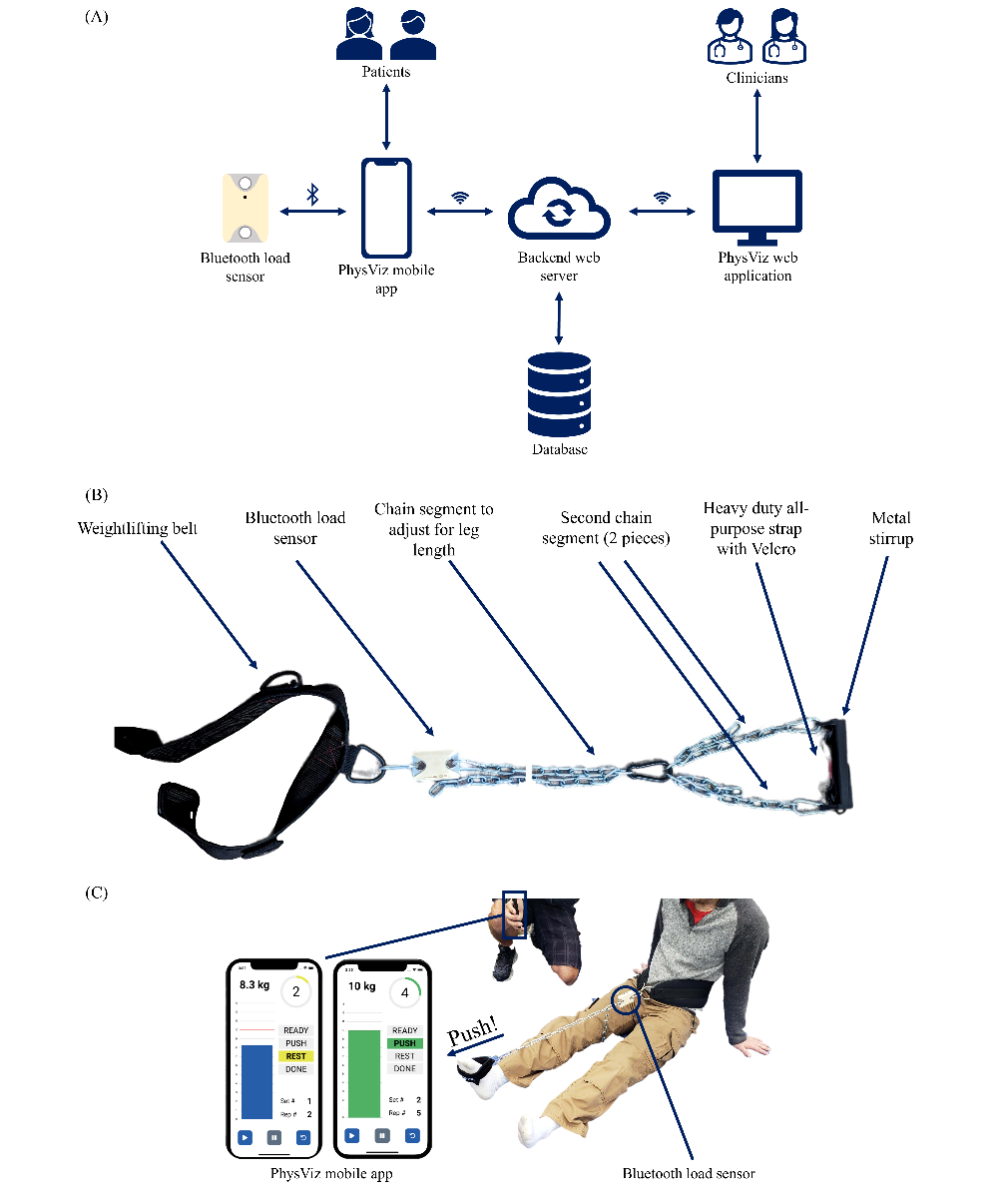
(A) System architecture diagram. (B) Current iteration of the PhysViz physical exercise tool. (C) Demonstration of intended system use with participant plantarflexing into the stirrup and imparting a load through the Achilles tendon. Participant simultaneously receives real-time biofeedback from the PhysViz mobile app.

On the basis of findings from the preceding stages and the operationalization of these findings in the logic model, we developed wireframe designs for both the PhysViz mobile app and web application (Figure 3 and Figure 4, respectively). The wireframe designs evolved based on circulation and iteration among the research team and were ultimately developed into working software packages for testing. In addition to the custom mobile app and web application, the physical exercise tool rounds out the PhysViz system architecture (Figure 5).

#### Load Sensor Selection and Testing

We selected the Progressor load sensor (Tindeq) for inclusion in the PhysViz system due to its superior performance across its measurement range compared with the Activ5 load sensor (Activbody). Complete findings are provided in the study by Merry et al [39].

#### PhysViz Validation

The findings indicate that the PhysViz is a valid and reliable tool for isometric plantar flexor MVC measurement; complete findings are provided in the study by Pratt et al [40]. In addition, according to the findings presented in the study by Schreiber et al [33], PhysViz biofeedback was found to enhance the execution of AT therapeutic exercises by lessening the percentage difference between recorded and prescribed load across a brief exercise protocol (ie, 2 sets of 10 five-second repetitions) compared to a nonbiofeedback condition.

#### Case Studies

Over the 12 weeks, participant 1, a male individual aged 35 years experiencing unilateral AT for 5 months, completed 27 therapeutic exercise sessions using the PhysViz. In this time, participant 1’s plantarflexion MVC on his affected side increased by 29% despite his pain score remaining unchanged.

Due to an additional sports-related injury impacting the affected side, participant 2, a male individual aged 40 years experiencing unilateral AT for 6 years, elected to discontinue the case study after 6 weeks. Over these 6 weeks, participant 2 completed 7 exercise sessions with the PhysViz. Only a single MVC test was recorded, precluding any inquiry into strength increases.

Participant 3, a female individual aged 57 years experiencing bilateral AT for 1 year, completed 8 exercise sessions with the PhysViz over the 12 weeks and increased her plantarflexion MVC by 20%. Participant 3’s pain score and self-reported pain tolerability was variable, with no clear trend.

Overall, information from the interviews showed that the PhysViz system was well received by all 3 participants. Specifically, the participants identified that compared to standard treatment options (ie, heel drops or calf raises), the real-time biofeedback was particularly useful because it promoted engagement in the rehabilitation process through the visualization of load intensity and the pacing of repetitions. Nevertheless, all 3 participants noted lower extremity pain while using the system, particularly in the knee, calf, and Achilles tendon. Despite reporting that the pain was tolerable, they expressed a desire to refine the positioning to mitigate the pain and improve overall comfort during use. Finally, the participants generally felt that the UI of the mobile app was adequate and accessible, although simplistic in comparison to other apps. If the plan is to disseminate the system beyond a research-based audience, the participants suggested improving the UI and adding additional features to bolster the somewhat rudimentary UX (eg, brief animations, reminders to complete exercises, and an in-app calendar showing completed and upcoming exercise sessions).

#### Multidisciplinary Roundtable Discussion

Of the 18 people invited to the discussion, 10 (56%) attended, including the research team members, external clinician-scientists, practicing clinicians (PTs and occupational therapists), people with lived AT experiences, and engineers. Overall, the group identified the main value proposition of the PhysViz system as being able to deliver high-magnitude loading precisely, either in the clinic or at home. Given the importance of loading intensity when managing AT, the invitees suggested that the PhysViz offered a useful alternative for patients who may not have time to go to the gym or lack access to the gym equipment often required to deliver the high loads needed to rehabilitate the tendon. Furthermore, the group identified that both PTs and individuals with AT stood to benefit from using the PhysViz: PTs can individualize and monitor care better when they have objective measurements such as those provided by the PhysViz; and individuals with AT are more likely to adhere to, and subsequently benefit from, treatment when they are able to see objective progress.

The group did identify that the design of the PhysViz physical exercise tool could be improved; in particular, the group sought to reduce the system complexity to make it accessible for more potential users. Specifically, the invitees reiterated the importance of modifying the design while maintaining flexibility in the system such that it can still be individualized to suit an array of patient needs (eg, body size, flexibility, and preexisting injuries). Depending on the revised design of the physical exercise tool, the invitees also suggested that the PhysViz could be used to help manage other lower limb tendinopathies (eg, patellar tendinopathy) and could be used as a general dynamometer to help augment care.

#### Patient and Clinician Usability Testing

According to the findings presented in the study by Merry et al [34], 15 people with AT and 4 PTs participated in the usability testing study. During the usability testing across the 8 tasks completed with the physical exercise tool and mobile app, people with AT encountered an average of 12 (SD 3) total usability issues. Most of the issues (102/183, 55.7%) were “severity 2—minor problems” according to the scale developed by Nielsen [45] and were most commonly issues associated with the “meaning of labels” (47/183, 25.7%), the “understanding of system instructions/error messages” (37/183, 20.2%), and the “visibility of system status” (31/183, 16.9%) according to the coding scheme postulated by Kushniruk and Patel [46]. During the clinician usability testing of the 3 tasks completed using the web application, PTs encountered an average of 3 (SD 3) total usability issues. Most of the issues (7/13, 54%) were “severity 2—minor problems” and were most commonly issues associated with the “meaning of labels” (8/13, 62%) and “navigation” (3/13, 23%).

During the debrief, 73% (11/15) of the people with AT stated that the PhysViz system was worth improving, with an additional 20% (3/15) stating that it is worth improving, provided research demonstrates its efficacy. Most of the participants with AT (13/15, 87%) stated that they would be willing to use a system such as the PhysViz during their AT rehabilitation. When asked to rate how helpful the PhysViz would be in supporting them to achieve their AT rehabilitation goals on a scale ranging from 0=*not helpful at all* to 10=*extremely helpful*, the system received a mean score of 7.2 (SD 1.2). By contrast, 50% (2/4) of the clinician participants stated that the PhysViz system was worth improving, with the other 50% (2/4) suggesting that it might be worth improving, provided changes were made to accommodate other musculoskeletal conditions besides AT.

Using the APEASE criteria, 25 proposed modifications were considered, and of these, 16 (64%) were deemed feasible for implementation in the next version of the PhysViz system (ie, having met all APEASE criteria). Cohen’s κ and prevalence-adjusted bias-adjusted κ values describing interrater agreement of the proposed PhysViz modifications were 0.39 (fair agreement) and 0.83 (almost perfect agreement), respectively.

## Discussion

### Principal Findings

#### Overview

This paper reports the formative development of the PhysViz, a novel remote rehabilitation system for people with AT using the FASTER. In addition to addressing the rationale and stakeholder needs underpinning the PhysViz, this project led to working prototypes of both the PhysViz physical exercise tool and the PhysViz software package that was tested by the potential end users (ie, both people with AT and PTs). Taken together, we think the development process outlined in this work represents a meaningful addition to the digital health landscape by providing a case study in systematic technology-based intervention development.

PhysViz development was framed within the development stage of the FASTER [25]. By comprehensively reviewing the literature and by engaging stakeholders through cross-sectional surveys and usability testing studies, this work leverages prior research knowledge, clinical insights and practice strategies, and patient-identified needs to improve the chances of intervention efficacy and uptake.

#### Empathize

The needs assessment, survey results [35], narrative review [17], and scoping review [38] collectively revealed critical insights into user expectations and preferences. Significantly, information was woven together from (1) research team members, (2) external stakeholders to whom we appealed directly, and (3) previous research, to inform system development in a robust way; for example, users expressed a demand for a versatile app compatible with both iOS and Android devices, with data-syncing capabilities for remote clinician monitoring. The emphasis on a simple and minimalistic UI aligns with the goal of inclusivity across different age groups and digital literacy levels. The incorporation of high-load tolerability and the use of the long sitting position in the design addressed specific biomechanical considerations highlighted in the narrative and scoping reviews.

#### Define and Ideate

The problem statement identified in the define stage highlighted the challenge of enabling individuals with AT to return to their activities quickly, emphasizing the importance of autonomy and objective feedback. The importance of autonomy and objective feedback identified aligns with past research [47], which found that personalized feedback can improve patient outcomes. This led to the formulation of a logic model, linking evidence-based features with behavioral and treatment outcomes. Taken together, both stages allowed the research team to critically reflect on the value proposition of the PhysViz system from the perspective of multiple end users, where it may fit along the treatment pathway, and how it may integrate into existing clinical workflows. Furthermore, mapping how different PhysViz system categories (eg, physical exercise tool and mobile app) could potentially address current treatment barriers helped mitigate potential design pitfalls such as unnecessary complexity and redundancy. Mapping also helped to delineate what features were needed in the PhysViz technologies (ie, physical exercise tool, mobile app, and web application) and what could be considered intervention support features (eg, the exercise protocol completed using the PhysViz, user training, and support for use).

#### Prototype and Test

##### Overview

Complexity was gradually built into the PhysViz system over months of development with the computer engineering student teams; varying levels of prototype fidelity were used to mock-up potential features and prove concepts in a timely manner. The resulting PhysViz system prototype and more specifically the UI and UX were then holistically evaluated toward the end of the prototype and test stages. Several features built into the PhysViz system prototype emerged directly from gaps in the literature and current standard care practices.

##### PhysViz UI and UX

The UI and UX of the PhysViz, tailored to address gaps and preferences identified in the emphasize stage, mirror recommendations from past literature that advocate for simplicity and intuitive design [48,49]. The needs assessment results underscored the importance of a simple and minimalistic UI, with a focus on ease of use for users across various age groups and digital literacy levels. Informed by the needs assessment and by the literature [45,50-53], we strategically designed the app and web portal with the goal of having simple screens and buttons as well as a predictable structure.

Moreover, the scoping review highlighted a significant gap in reporting related to adherence, compliance, and fidelity to prescribed exercises in AT interventions [38]. This poor reporting of adherence, compliance, and fidelity is also reflected in the existing literature [54,55], highlighting a need for improved monitoring systems. The UI and UX of the PhysViz addresses this gap by incorporating features for automated tracking of exercise sessions (ie, there is no “save/upload data” button present; the system does this automatically), accompanied by intuitive visual cues and real-time biofeedback in the form of repetition timing and load intensity monitoring. The various forms of exercise cueing and data visualization aim to empower users to increase their adherence, fostering a more engaging and motivating rehabilitation experience. The case studies underscored participants’ positive experiences with the PhysViz UI and UX, particularly appreciating aspects such as the color change effect when “target load” intensity was reached, as well as the pacing of repetitions. These results are further supported by previous literature, which highlights the utility of biofeedback to enhance user engagement and adherence [56,57].

During usability testing, most of the usability issues encountered by people with AT related to the “meaning of labels” (47/183, 25.7%), “understanding of system instructions” (37/183, 20.2%), and “visibility of system status” (31/183, 16.9%) [34]. When assessing the web application, most of the usability issues PTs faced were associated with the “meaning of labels” (8/13, 62%) and “navigation” (3/13, 23%). Usability testing results such as these will help inform further UI and UX iteration to improve the PhysViz by specifically identifying problematic features or areas according to end users.

In the case studies, participants generally perceived the UI of the mobile app as adequate and accessible, although they acknowledged its simplicity compared to other apps. Notably, suggestions emerged for enhancing the UI, particularly if the goal is to extend the reach of the PhysViz system beyond a research-based audience. Recommendations included incorporating brief animations, reminders for completing exercises, and an in-app calendar displaying completed and upcoming exercise sessions, aligning with behavior change literature [58,59]. These suggestions, if implemented, could address the reported simplicity of the UI and enhance the overall UX, making the PhysViz more accessible to a wider audience.

##### Biofeedback

Biofeedback emerged as a promising feature with the potential to address several gaps in existing treatment strategies. The need for precision loading, a key consideration highlighted in the narrative review [17], is directly addressed through real-time biofeedback in the form of load intensity monitoring, allowing users to achieve and maintain high-magnitude loading with greater accuracy. The results from the validation studies substantiate this claim, demonstrating that the PhysViz system is a valid and reliable tool for isometric plantar flexor MVC measurement and that biofeedback is a useful feature for promoting the accurate execution of exercise parameters such as load intensity [33,40]. Given the lack of reporting on exercise fidelity identified in the scoping review, biofeedback and the automated tracking of exercise sessions incorporated in the PhysViz may enhance program fidelity for individuals with AT while also facilitating transparent and automated reporting for clinicians.

The importance of transparent reporting was also identified in the surveys, which revealed a perceived lack of patient compliance among PTs [35]. Biofeedback has the potential to bridge this gap by supplying both people with AT and clinicians with objective, measurable data, fostering a collaborative approach to rehabilitation and improving overall treatment compliance. In the case studies, participants commended the real-time biofeedback provided by the PhysViz, citing its utility in promoting engagement through the visualization of load intensity and the pacing of repetitions. Moreover, insights from the multidisciplinary roundtable discussion, including perspectives from clinicians and individuals with AT, underscored the value proposition of the PhysViz in delivering high-magnitude loading precisely, thus enhancing its potential to address compliance challenges. Usability testing further supported the positive impact of biofeedback, with individuals expressing willingness to use the PhysViz during their rehabilitation, highlighting its potential to improve overall treatment compliance [34].

This multifaceted evidence stemming from both sets of end users surveyed [35] and existing literature [17,38] highlights the potential for precision loading and the need for improved therapeutic exercise engagement among people with AT. The findings were substantiated during the prototype and test stages of this work, including the validation studies [33,40], case studies, roundtable discussions, and usability testing [34]. Together, this body of evidence supports the notion that the biofeedback offered by the PhysViz serves as a valuable instrument for strengthening collaborative rehabilitation initiatives and promoting increased fidelity and, potentially, engagement with therapeutic exercises for AT rehabilitation.

### Comparison to Previous Work

This work aligns with and builds upon existing literature regarding telehealth development, particularly within the context of human-centered design for health care interventions. Emerging research suggests that integrating human-centered design into telehealth research results in more usable, acceptable, and effective health care interventions [60,61]; however, there is significant underreporting and a lack of guidance in the development of sustainable health care apps [62], and much of the telehealth app research focuses more on laboratory and field evaluations than on the design and development phases [63].

Our work addresses these gaps by emphasizing the early stages of the development process, specifically the empathize and define stages within the FASTER, where thorough needs assessments and stakeholder engagement initiatives were conducted. This approach allowed us to prioritize end-user needs and design features that are both practical and meaningful for the target population. By focusing on the development phase, we ensured that the PhysViz system was not only functional but also aligned with the preferences and constraints of potential end users. In addition, our iterative development process, which included usability testing and multidisciplinary discussions, contributed to creating a more sustainable telehealth solution that can adapt to the evolving needs of its end users. This proactive approach contrasts with much of the existing telehealth research, which often underrepresents the importance of early-stage design and development in favor of later-stage evaluations [63].

A recent review of telehealth app development highlighted that lengthy development times and the use of limited qualitative research methods are often cited as constraints associated with the long-term efficacy of the apps [63]. Telehealth app development frequently uses interviews and focus groups [63], and while these methods are effective for gathering direct feedback from end users, they have been criticized for their limited ability to elicit tacit knowledge [64]. While our work did use these methods, we also relied on multiple smaller-scale and time-efficient studies, using diverse methods to capture both explicit insights (through the surveys and multidisciplinary roundtable discussion) and tacit knowledge (through case studies and usability testing). This mixed methods approach not only allowed us to gather comprehensive data on end-user needs and preferences but also facilitated the iterative refinement of the PhysViz system, ensuring that it was both user centered and responsive to the complexities of real-world use. By integrating feedback from a variety of stakeholders—patients, clinicians, and engineers—we were able to design a telerehabilitation solution that is more likely to be effective, sustainable, and widely accepted in clinical practice. This approach highlights the importance of combining traditional qualitative methods with more agile techniques to enhance the development and adoption of telehealth technologies.

While telehealth apps are often developed with the inclusion of end users, this engagement tends to be limited to a single user group such as patients or clinicians [65]. By contrast, our work involved multiple end-user groups throughout the development process, including people with AT as well as clinicians and stakeholders responsible for the implementation and dissemination of the PhysViz. This comprehensive approach allowed for a more holistic understanding of the diverse needs, preferences, and challenges associated with both the use and the integration of the system. By engaging these varied perspectives, we were able to design a more adaptable and user-friendly telehealth solution that not only meets the therapeutic needs of patients but also aligns with the practical and logistical requirements of clinicians and health care systems. This multistakeholder engagement is critical in ensuring that the resulting technology is effective, sustainable, and capable of being seamlessly integrated into routine clinical practice.

### Strengths and Implications

This work represents one of the first uses of the FASTER development phase in developing a remote rehabilitation intervention (including a physical exercise tool, a mobile app, and a web application). The collaborative and multidisciplinary nature of this work is a strength, particularly in integrating perspectives from people with the condition of interest and clinician partners throughout the timely and rigorous development of the PhysViz system prototype. Involving a diverse team, including those with lived experiences, has not only enriched the development process but also ensured that the resulting intervention is attuned to the real-world needs and preferences of end users. This inclusive approach enhances the credibility and applicability of the system and serves as a model for future technology-based rehabilitation development efforts.

Another notable strength lies in the diversity of study types used. Tailoring study designs to suit development needs is a core feature of the FASTER, which suggests a variety of methods for informing and evaluating the proposed intervention. By incorporating narrative and scoping reviews, case studies, and both qualitative and quantitative data, this research amalgamation effectively addresses the limitations inherent in a single-study approach. This comprehensive methodological approach allows for a more nuanced understanding of the intervention’s usability according to end users and potential effectiveness in managing AT through the mobilization of high-magnitude precision loading. It also contributes to a more holistic assessment of the PhysViz system, capturing a range of perspectives and insights that might be overlooked in a single-study design.

Furthermore, the systematic identification of theory-based techniques, such as the integration of the COM-B model, adds a layer of robustness to the intervention development process. By aligning the PhysViz system with behavior change theory, we enhance our understanding of the underlying mechanisms and pave the way for more targeted and potentially effective interventions. This methodological rigor ensures that the developed intervention is technologically sound and theoretically grounded, increasing its potential for successful implementation in real-world rehabilitation settings.

### Future Directions

The positive reception from users and clinicians underscores the potential impact of the PhysViz system; however, the identified areas for improvement, such as improving consistency and intuitive navigation, should guide future iterations. More specifically, mobile app clarity can be improved by adding a designated “Home” button within the app UI to anchor users, adding an in-app back button, and avoiding potentially confusing words such as “tare.” Further efforts seem necessary to enhance the UI and UX of the biofeedback interface to present users with crucial information while preventing information overload. Furthermore, the potential extension of the PhysViz to manage other tendinopathies and its use as a general purpose dynamometer offer exciting possibilities for broader clinical applications. Future research should explore these potential applications and assess the feasibility and effectiveness of the PhysViz in diverse clinical and home-based settings.

### Limitations

This study has several limitations. First, the PhysViz development detailed in this work constitutes only 1 cycle through the FASTER development phase; further iterations of the system are recommended before proceeding to FASTER phase 2 (progressive usability and feasibility evaluation). In addition, because the FASTER provides little guidance regarding when it is appropriate to move between phases, some research and development discretion is needed. Second, long-term exposure to the PhysViz system by end users (people with AT or clinicians) was limited. Three people with AT completed case studies, although the use of the system during these studies was relatively low, limiting the comprehensiveness of feedback. Moreover, the lack of any long-duration (ie, >1 session) testing by the clinicians makes it challenging to comment on their opinions of the PhysViz within the context of their working environment. Although the clinicians were asked how they might integrate the PhysViz into their practice, the study did not specifically explore potential logistical or legal challenges they might face, which presents an area for future research. Third, working with the computer engineering student teams helped control costs when completing development and provided students with experiential learning opportunities; however, because of the course structure, coupled with other commitments, the students were limited in their development capacity. Furthermore, we did not have a software developer actively working on the project. As a result, development iterations took a significant amount of time to implement, limiting the rapid prototyping and feedback cycle typical of engineering design. We tried to circumvent this by using wireframe models and other low-resolution strategies where possible.

### Conclusions

This paper describes the development process of the novel PhysViz system, an exercise-based remote rehabilitation system for AT management. This is one of the first studies to describe using the FASTER for technology-based intervention development; by following the FASTER development phase guidelines, the PhysViz incorporates past evidence, end-user needs and opinions, and behavior change science. Our goal is that the PhysViz will be used to improve the current knowledge of appropriate therapeutic exercise dosing for tendinopathic tissue rehabilitation as well as improve the pain, functional status, and activity of people with chronic AT. Future iterations of the PhysViz may be adapted for other home-based treatment strategies that provide user autonomy with remote clinical supervision, thereby decreasing in-clinic time and associated costs.

## Appendix

Multimedia Appendix 1 Detailed logic models.

## Abbreviations

APEASE: affordability, practicability, effectiveness, acceptability, side effects and safety, and equity
AT: Achilles tendinopathy
COM-B: capability, opportunity, motivation, and behavior
FASTER: Framework for Accelerated and Systematic Technology-Based Intervention Development and Evaluation Research
MVC: maximal voluntary contraction
PT: physical therapist
UI: user interface
UX: user experience
