# Supplementary material for: An Exercise-Based Precision Medicine Tool and Smartphone App for Managing Achilles Tendinopathy (the 'PhysViz' System): User-Centered Development Study
Source: JMIR Hum Factors. 2024 Nov 13;11:e57873. doi: 10.2196/57873 (PMC11602768; doi:10.2196/57873)
Supplement: Multimedia Appendix 1 [file humanfactors_v11i1e57873_app1.docx]

## Multimedia Appendix 1

Detailed logic models


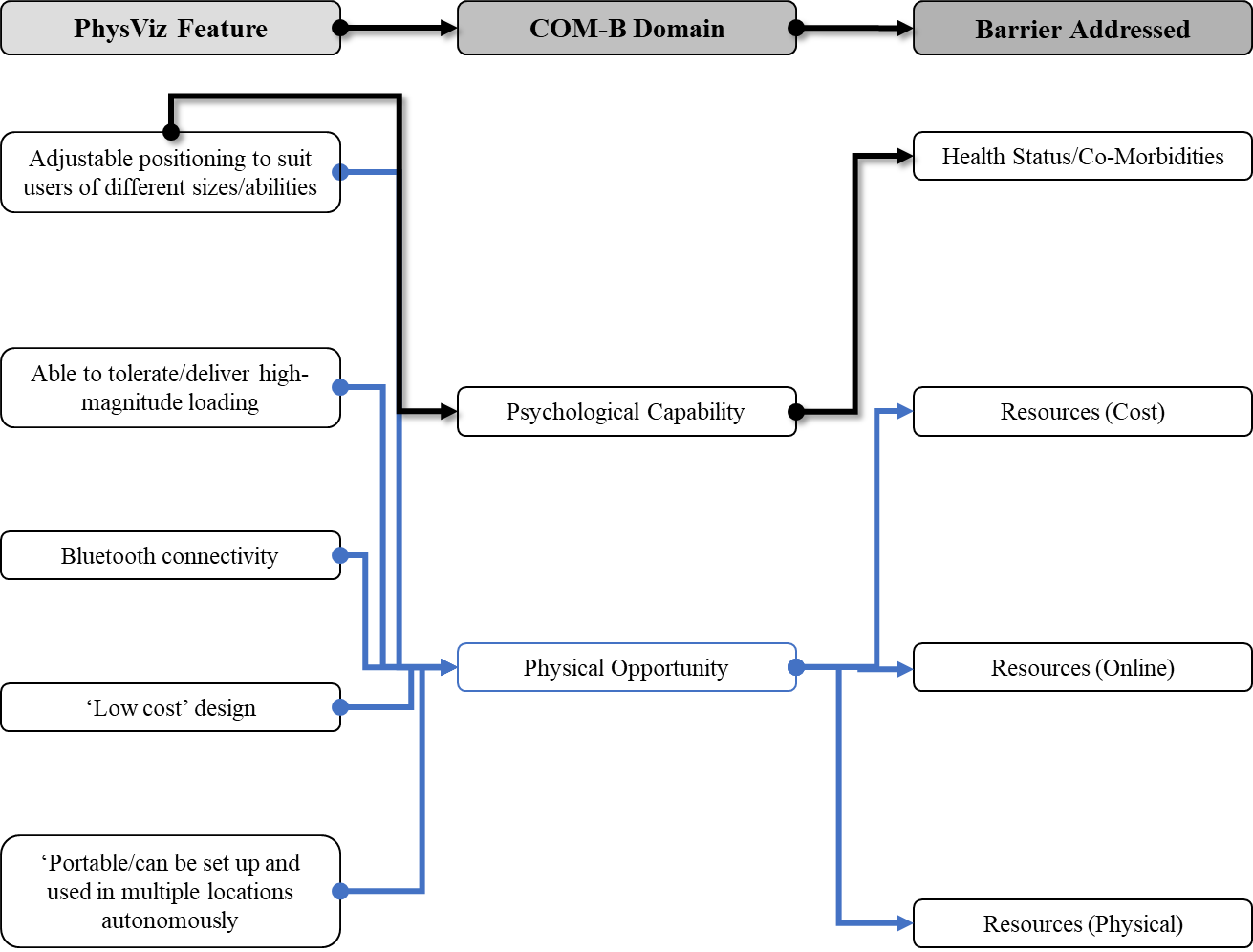


PhysViz development detailed logic model – Physical Exercise Tool


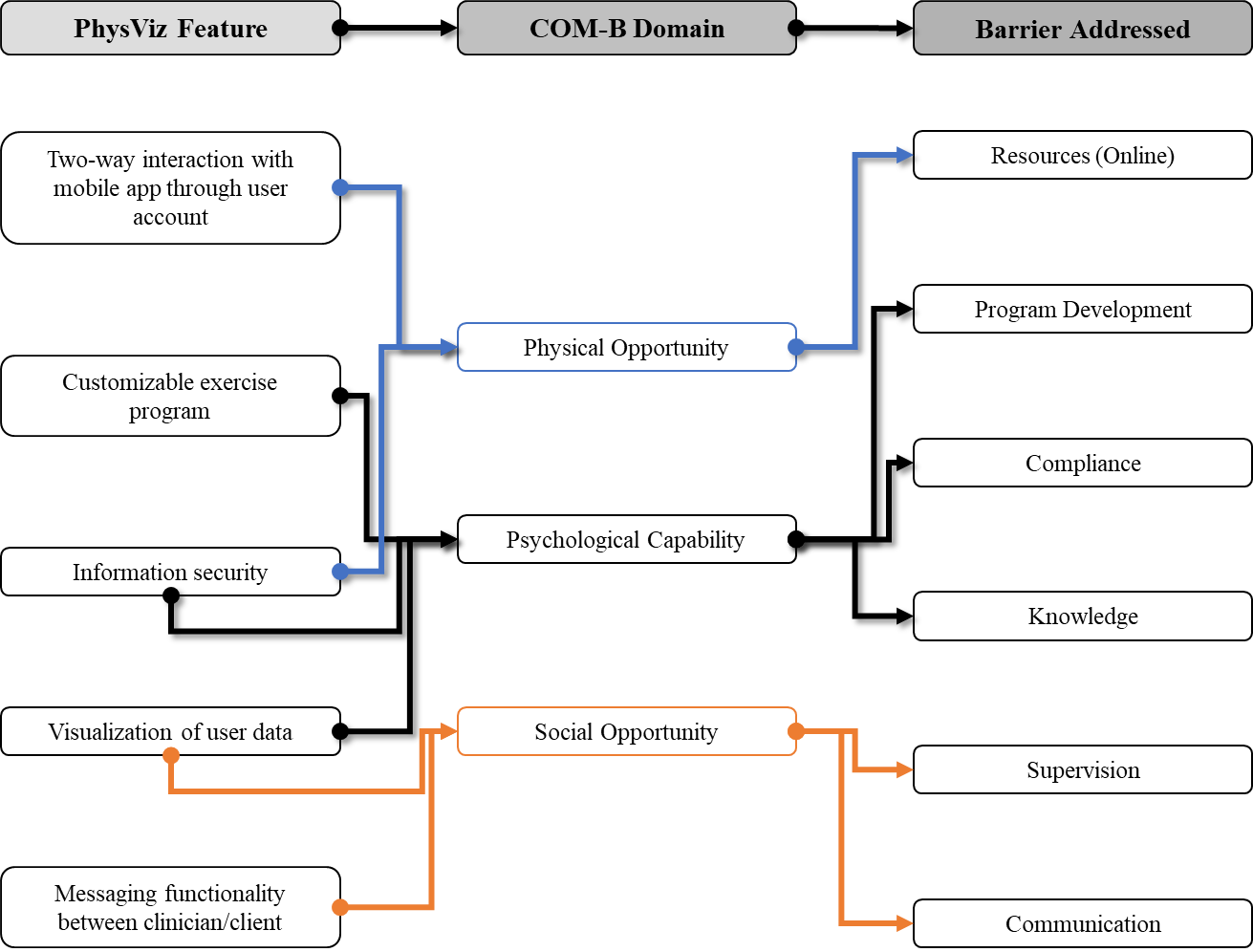


PhysViz development detailed logic model – Web Application


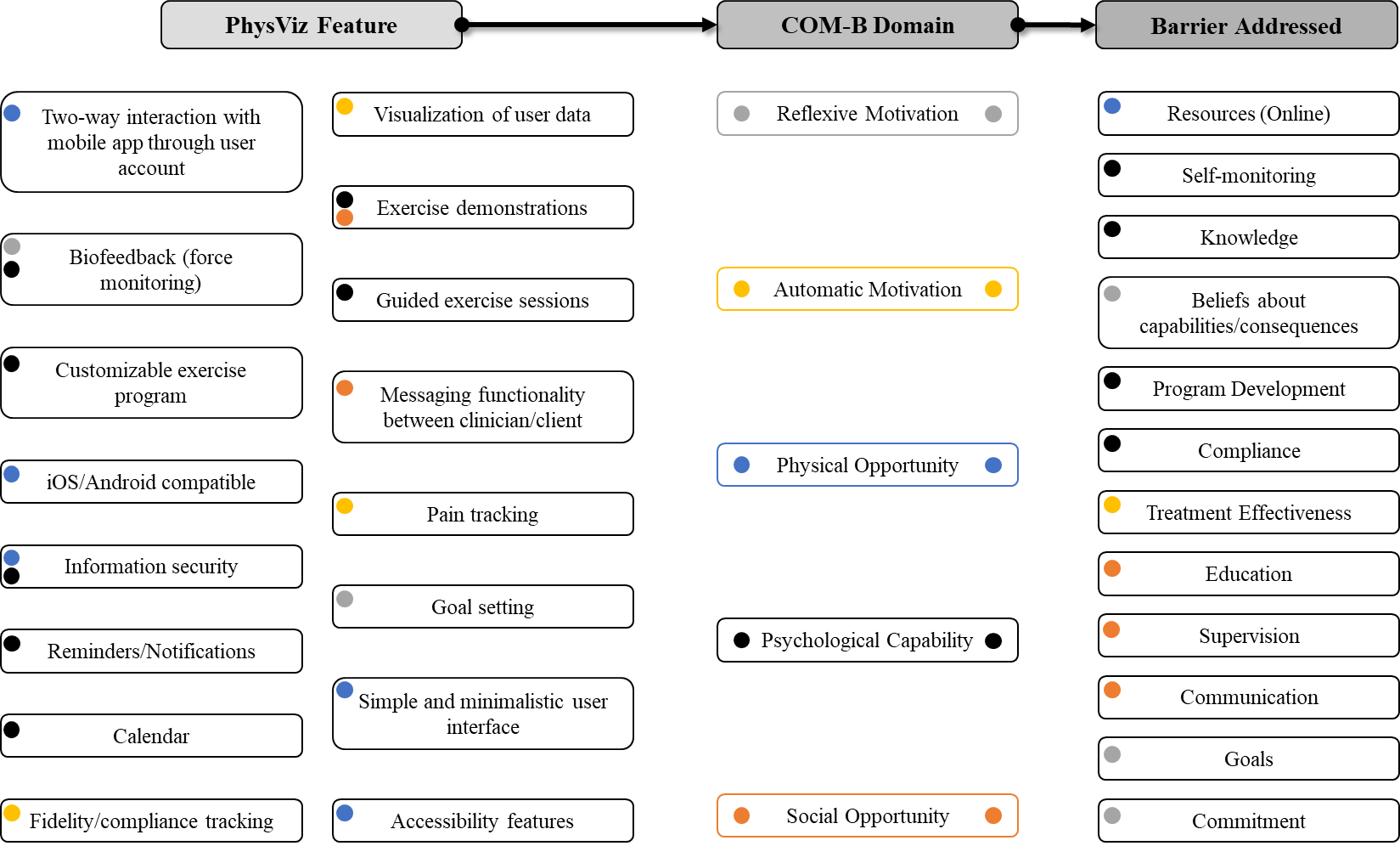


PhysViz development detailed logic model – Mobile Application


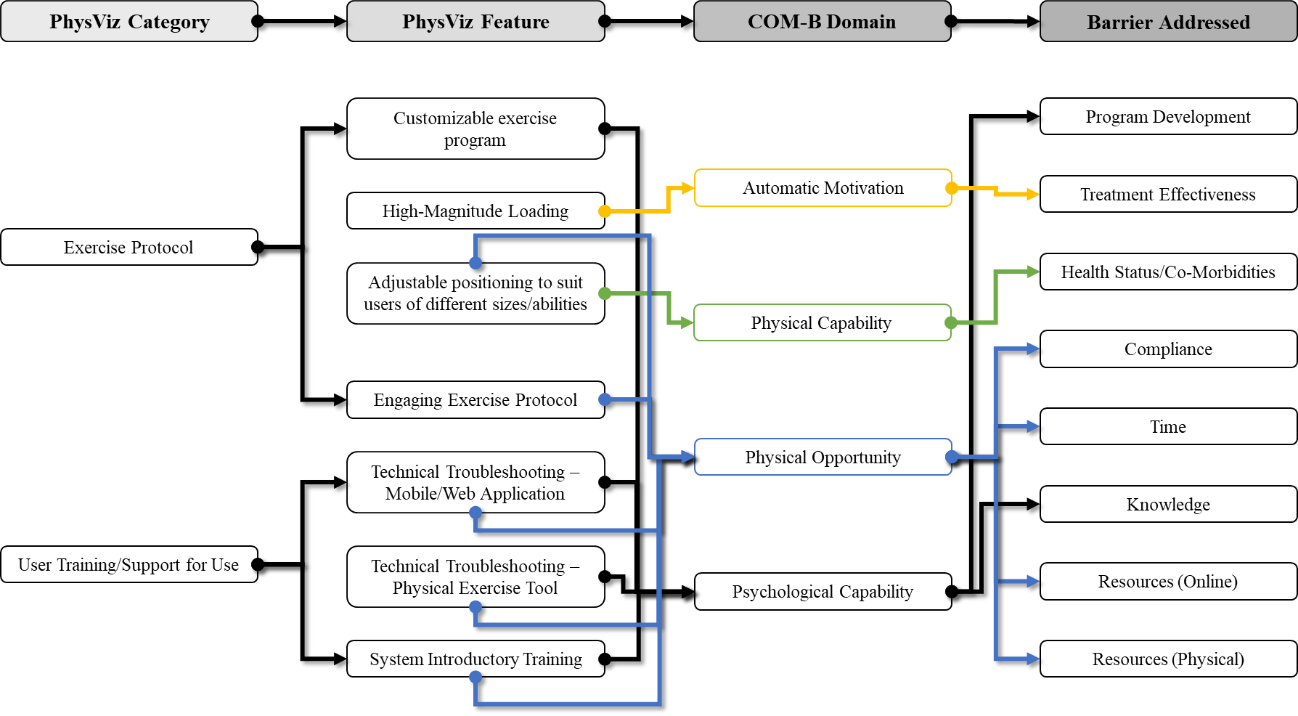


PhysViz development detailed logic model – Support Features
